# Diagnostic Accuracy of Oxygen Desaturation Index for Sleep-Disordered Breathing in Patients With Diabetes

**DOI:** 10.3389/fendo.2021.598470

**Published:** 2021-03-09

**Authors:** Lihong Chen, Weiwei Tang, Chun Wang, Dawei Chen, Yun Gao, Wanxia Ma, Panpan Zha, Fei Lei, Xiangdong Tang, Xingwu Ran

**Affiliations:** ^1^ Diabetic Foot Care Center, Department of Endocrinology and Metabolism, West China Hospital, Sichuan University, Chengdu, China; ^2^ Sleep Medicine Center, Mental Health Center, Translational Neuroscience Center, and State Key Laboratory of Biotherapy, West China Hospital, Sichuan University, Chengdu, China

**Keywords:** sleep disordered breathing (obstructive/central sleep apnea), diabetes, oxygen desaturation index (ODI), polysomnogram (PSG), diagnostic accuracy

## Abstract

**Background:**

Polysomnography (PSG) is the gold standard for diagnosis of sleep-disordered breathing (SDB). But it is impractical to perform PSG in all patients with diabetes. The objective was to develop a clinically easy-to-use prediction model to diagnosis SDB in patients with diabetes.

**Methods:**

A total of 440 patients with diabetes were recruited and underwent overnight PSG at West China Hospital. Prediction algorithms were based on oxygen desaturation index (ODI) and other variables, including sex, age, body mass index, Epworth score, mean oxygen saturation, and total sleep time. Two phase approach was employed to derivate and validate the models.

**Results:**

ODI was strongly correlated with apnea-hypopnea index (AHI) (r_s_ = 0.941). In the derivation phase, the single cutoff model with ODI was selected, with area under the receiver operating characteristic curve (AUC) of 0.956 (95%CI 0.917–0.994), 0.962 (95%CI 0.943–0.981), and 0.976 (95%CI 0.956–0.996) for predicting AHI ≥5/h, ≥15/h, and ≥30/h, respectively. We identified the cutoff of ODI 5/h, 15/h, and 25/h, as having important predictive value for AHI ≥5/h, ≥15/h, and ≥30/h, respectively. In the validation phase, the AUC of ODI was 0.941 (95%CI 0.904–0.978), 0.969 (95%CI 0.969–0.991), and 0.949 (95%CI 0.915–0.983) for predicting AHI ≥5/h, ≥15/h, and ≥30/h, respectively. The sensitivity of ODI ≥5/h, ≥15/h, and ≥25/h was 92%, 90%, and 93%, respectively, while the specificity was 73%, 89%, and 85%, respectively.

**Conclusions:**

ODI is a sensitive and specific tool to predict SDB in patients with diabetes.

## Introduction

Sleep-disordered breathing (SDB) is characterized by episodic sleep state dependent collapse of the upper airway, resulting in periodic reductions or cessations in ventilation, with consequent hypoxia, hypercapnia, or arousals from sleep (1). SDB is a common disease with a prevalence of 3% among women and 10% among men 30 to 49 years of age and 9% among women and 17% among men 50 to 70 years of age in the general population (2). It has been estimated that there is a high prevalence of undiagnosed SDB in patients with type 2 diabetes (24–86%) (3, 4). Moreover, the prevalence of moderate-to-severe SDB was more than 50% in patients with diabetic foot ulcers (DFUs) (5). Patients with SDB are more likely to have hypertension, stroke, heart failure, and depression (6). Furthermore, SDB is associated with increased risk of complications of diabetes, such as diabetic polyneuropathy, peripheral artery disease, and ulcer development (7, 8). Thus, early diagnosis of SDB in patients with diabetes is essential.

The overnight polysomnography (PSG) is the gold standard for the diagnosis of SDB. However, PSG is a time-consuming and costly procedure. In addition, it is a rather uncomfortable experience for some patients. In our previous study, nearly one-third of the patients with diabetic foot ulcers did not complete the process of the test because of the uncomfortable experience (5). Home sleep apnea testing can also be used for diagnosing sleep apnea in uncomplicated patients. However, patients with diabetes often suffer from diabetes related complications, such as diabetic neuropathy, which might compromise the result of home sleep apnea testing (9). Thus, it is necessary to develop an easy-to-use method based on clinical features and accessible metrics to predict the likelihood of SDB.

Many screening tools have been developed, such as the often-utilized Epworth Sleepiness Scale and “STOP-BANG”. However, they have minimal predictive value for SDB (10–12). Multistep screening approaches, such as using a questionnaire or prediction tool followed by overnight home-based testing, did not have adequate discrimination (13). Machine learning, such as Neural Network and Support Vector Machine, have been utilized to predict the SDB using clinical features (e.g. age, sex, and smoking) and physiologic measurements (e.g. blood pressure, overnight pulse oximetry, and pulmonary function) (14, 15). The predictive performance varied among studies with sensitivity from 66% to 100%, and specificity from 30.8% to 76.2% to predict AHI ≥5/h. However, the predictive models derived from machine learning are sometimes difficult to understand and hard to use in clinical practice (16). Furthermore, most studies were carried out at sleep centers in the general population with possible SDB. Given the high prevalence of SDB in patients with diabetes and the bidirectional association between SDB and diabetes (17), it is crucial to validate the criminative performance of models in patients with diabetes. However, there was lack of study that investigated the predictive models in patients with diabetes.

SDB is often accompanied with hypoxemia. Oxymetric measures can be extracted from pulse oximetry, including mean oxygen saturation, percentage of time with oxygen saturation < 90%, and oxygen desaturation index (ODI). A systematic review evaluated the diagnostic accuracy of ODI for SDB, significant heterogeneity exists among studies with sensitivities ranged from 32% to 98.5%, while specificities ranged from 47.7% to 98% (18). Furthermore, studies have revealed that hypoxemia, rather than AHI, were well associated with cardiovascular risk (19, 20). Till now, no study has been published to evaluate the diagnostic performance of ODI in patients with diabetes. The high prevalence of SDB in patients with diabetes and associated diabetic complications such as diabetic neuropathy might influence the predictive accuracy of ODI (4, 21). Thus, it is imperative to evaluate the diagnostic performance of ODI in patients with diabetes.

The purpose of the study was to develop a model based on ODI to identify patients with SDB at three AHI cutoffs (≥5/h, ≥15/h, and ≥30/h) in patients with diabetes.

## Methods

### Patients

In this study, a total of 440 patients with diabetes were recruited and underwent overnight PSG at the ward of Endocrinology and Metabolism of West China Hospital, Sichuan University between July 2013 and June 2019. Patients who had a diagnosis of specific types of diabetes due to other causes, such as Cushing syndrome, Acromegaly, and those who were being treated with corticosteroids, immunosuppressive drugs, or chemotherapy, were excluded. Patients who experienced severe hypoglycemia were excluded. No participant had prior diagnosis of sleep disorders or was being treated with continuous positive airway pressure. This study was approved by the Biomedical Research Ethics Committee of West China Hospital of Sichuan University, and all participants provided written informed consent.

Demographics, comorbidities, and laboratory data were obtained from the patient’s medical record at the date of admission. The demographics included age, sex, smoking, BMI, duration of diabetes. Daytime sleepiness was assessed by the Epworth Sleepiness Scale (ESS), with excessive daytime sleepiness defined as ESS ≥10 (22).

### Procedures

An overnight unattended portable PSG recording (Somté, Compumedics, Australia) was performed inpatient, five to seven days following hospitalization. Subjects were allowed to sleep based on their habitual sleep time. The apparatus included electroencephalography, bilateral electrooculography, electrocardiography, electromyography (submental and anterior tibialis), nasal pressure and thermal airflow, thoracoabdominal movements, and pulse oxygen saturation. Sleep recordings were scored by certified sleep technologists, who were blind to any diagnosis.

Respiratory events were manually scored according to 2012 AASM recommendations (23). Apnea was defined as a drop of at least 90% of airflow from baseline lasting 10 s or longer. Hypopnea was defined as a reduced airflow of 30% from baseline for more than 10 s, in association with either a 3% oxygen desaturation or an arousal. AHI was calculated as number of apnea and hypopnea events per hour of sleep. SDB severity was graded as follows: normal (AHI <5 events/hour), mild (AHI 5–14.9 events/hour), moderate (AHI 15–29.9 events/hour), and severe (AHI ≥30 events/hour). The three cutoffs of AHI (≥5/h, ≥15/h, and ≥30/h) for SDB were regarded as the reference standard.

Because oximetry is one of the PSG channels, we derived oxymetric measures, including mean oxygen saturation and ODI (number of events per hour in which oxygen saturation decreased by ≥3% from baseline), from the PSG test.

### Statistical Analysis

We summarized data as either the number of patients (%), mean (SD), or median (IQR). We did the bivariate analysis with Chi-square test, Student’s t test, or Mann-Whitney U test. Random split-sample function was used to randomly split the full data set into two data sets: 2/3 of the patients was used for derivation (derivation data set), and the remaining 1/3 was used for validation (validation data set).

In the derivation phase, spearman correlation between ODI and AHI was analyzed. Scatter plot and simple linear regression between ODI and AHI were depicted and explored. Prediction algorithms were based on oxygen desaturation index (ODI) and other variables, including sex, age, body mass index, Epworth score, mean oxygen saturation, and total sleep time. Three models were applied for each prediction (≥5/h, ≥15/h, and ≥30/h): a model with ODI; a model with ODI and Epworth score; a model with sex, age, BMI, Epworth score, ODI, oxygen saturation, and total sleep time (full model). K-Fold Cross-Validation (k=10) was used to generate the model. DeLong’s test was carried out to compare the area under the receiver operating characteristic curve (AUC) among three models. The model with ODI alone was selected because the AUCs were similar among three models. The Youden’s index was employed to find the optimal cutoff. McNemar’s test was employed to compare the sensitivities and specificities between the derived optimal cutoff and a simplified cutoff point (5/h, 15/h, and 25/h) (24). The cutoff points of 5/h, 15/h, and 25/h for predicting AHI ≥5/h, ≥15/h, and ≥30/h was preferred for reasons of simplicity and ease of use.

For validation, sensitivity, specificity, negative predictive value, positive predictive value, and AUC, with corresponding 95% confidence intervals, were assessed at chosen cutoffs in the validation data set. Predictive performance was also estimated in the full data set. Statistical analysis was performed with Stata version 13 (Stata Corp.). A p value below 0.05 was considered statistically significant.

## Results

The baseline characteristics are listed in **Table 1** and **Supplementary Table S1** (derivation data set) and **Table S2** (validation data set) (categorized according to SDB severity). The average age was 58 ± 14 (range 18–89) years, and 66% of patients were men. Most of the patients included were patients with type 2 diabetes, with only three cases of type 1 diabetes. The median duration of diabetes was 9 (3–15) years, with a mean glycated hemoglobin (HbA1c) level of 8.8 ± 2.2%. The median AHI was 23.9 (9.7–41.7)/h, with a prevalence of 90.5%, 62.7%, and 39% for AHI≥5/h, ≥15/h, and ≥30/h, respectively. The clinical demographics and comorbidities were similar between patients in the validation group and those in the derivation group. Overall, the patients above the AHI cutoff had lower mean oxygen saturation, higher T90%, and higher ODI, while there was no significant difference in Epworth score.

**Table 1:** Baseline characteristics of patients in the full data set.

	Total	Derivation dataset	Validation dataset	P value
	N = 440	N = 293	N = 147	
Male sex	290 (66%)	191 (65%)	99 (67%)	0.652
Age, y/o	58 ± 14	57 ± 15	59 ± 14	0.189
BMI, kg/m^2^	25.9 ± 5.9	26.0 ± 6.1	25.8 ± 5.7	0.692
Smoking	210 (48%)	137 (47%)	73 (50%)	0.565
Duration of diabetes, y	9 (3–15)	9 (3–16)	10 (3–15)	0.646
Hypertension	292 (73%)	190 (65%)	102 (69%)	0.342
Ischemic heart disease	73 (17%)	46 (16%)	27 (18%)	0.478
Peripheral artery disease	77 (18%)	49 (17%)	28 (19%)	0.545
Retinopathy	117 (27%)	78 (26%)	39 (27%)	0.984
Peripheral nervous disease	282 (64%)	188 (64%)	94 (64%)	0.964
Diabetic kidney disease	143 (33%)	92 (31%)	51 (35%)	0.486
Diabetic foot ulcer	110 (25%)	79 (27%)	31 (21%)	0.180
HbA1c, %	8.8 ± 2.2	8.7 ± 2.2	8.9 ± 2.2	0.493
Epworth score	6 (2–11)	6 (2–10)	6 (3–11)	0.423
>10	140 (32%)	89 (30%)	51 (35%)	0.359
AHI,/h	23.9 (9.7–41.7)	24.0 (9.7–43.6)	21.0 (9.8–38)	0.713
Total sleep time, h	6.5 ± 1.6	6.5 ± 1.6	6.4 ± 1.6	0.512
Sleep efficiency, %	75 ± 15	74 ± 15	75 ± 15	0.485
T90%, %	4 (1–16)	4 (0–16)	3 (0–15)	0.520
Mean oxygen saturation, %	93 ± 4	93 ± 4	94 ± 3	0.170
ODI,/h	21.7 (10.9–39.9)	22.5 (10.9–42.4)	21.3 (10.9–35.2)	0.324

Data are shown as mean (SD), median (quartile), or count (%).

BMI, body mass index; AHI, apnea-hypopnea index; HbA1c, hemoglobin A1c; T90%, percentage of time with oxygen saturation < 90%; ODI, oxygen desaturation index.

### Derivation Phase

**Figure 1:**
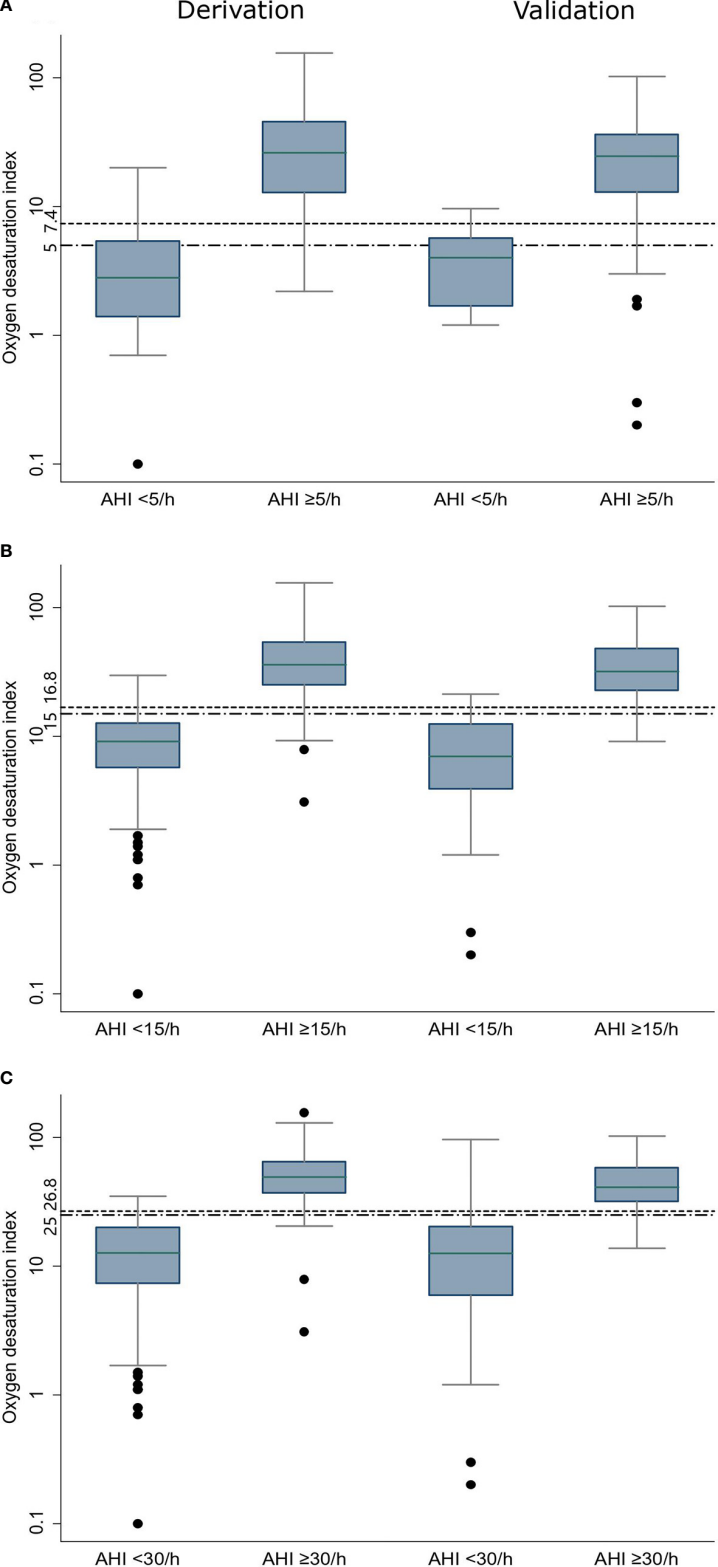
Oxygen desaturation index in the derivation and validation data sets at three AHI cutoffs (**A**: AHI ≥5/h, **B**: AHI ≥15/h, and **C**: AHI ≥30/h). The bottom and top boundaries of each box represent the upper and lower quartiles, the lines within the box represent the median, the whiskers represent values that are 1.5 times the interquartile range, the dots represent values outside the range, and the horizontal lines represents the cutoff point of Oxygen desaturation index. AHI: apnea-hypopnea index.

The median ODI was 26.2/h, 36.1/h, and 49.3/h, respectively, much higher among patients with AHI ≥5/h, ≥15/h, and ≥30/h. For the corresponding patients who had an AHI value below the cutoffs, the median ODI was 2.8/h, 9.1/h, and 12.7/h, respectively (**Figure 1**). There was a strong correlation between ODI and AHI, with Spearman coefficient of 0.941. The relationship between ODI and AHI is further depicted in **Supplementary Figure S1**. Thus, three models based on ODI were set up to predict SDB.

The AUCs were similar in the three models for predicting SDB (**Supplementary Table S3**). Given the simplicity, the model which included only ODI was selected. The cutoffs identified using Youden’s index in the model were 7.4, 16.8, and 26.8. For simplicity, 5, 15, and 25 were preferred for clinical usage. The comparison of derived cutoffs and simplified cutoffs were shown in **Supplementary Table S4**. Overall, the simplified cutoffs had higher sensitivity, but still retained good specificity.

### Validation Phase

In the validation group, the median ODI was 24.6/h, 31.8/h, and 41.1/h, respectively, much higher among patients with AHI ≥5/h, ≥15/h, and ≥30/h. For the corresponding patients who had an AHI value below the cutoffs, the median ODI was 4.0/h, 7.0/h, and 12.6/h, respectively (**Figure 1**).

**Figure 2:**
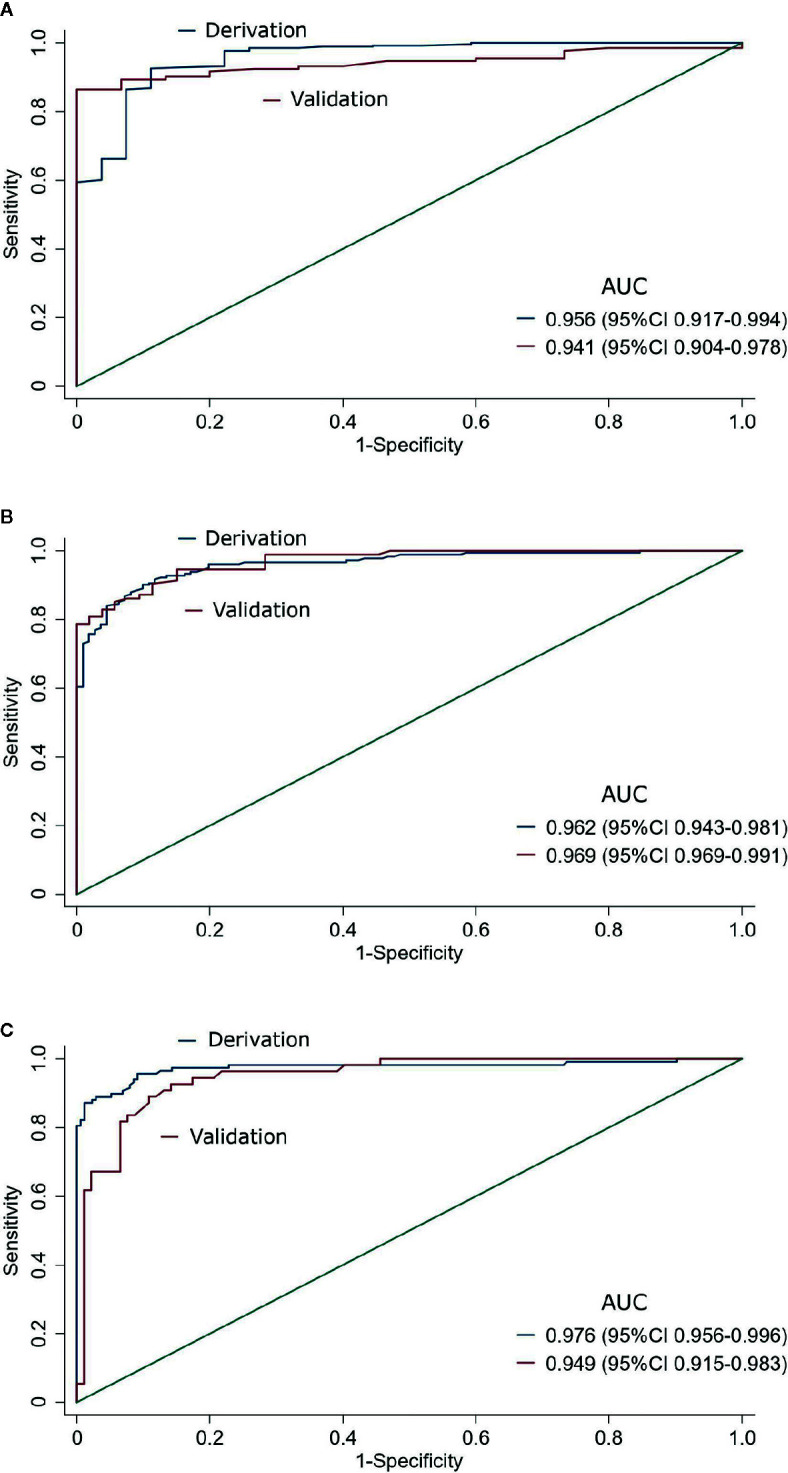
Predictive performance of Oxygen desaturation index for sleep-disordered breathing in the derivation and validation data sets at three AHI cutoffs (**A**: AHI ≥5/h, **B**: AHI ≥15/h, and **C**: AHI ≥30/h). AUC: the area under the receiver operating characteristic curve.

The discriminative ability of ODI remains good consistently for all three AHI cutoffs. The AUC was 0.941 (95% CI, 0.904–0.978), 0.969 (95% CI, 0.969–0.991), and 0.949 (95% CI, 0.915–0.983) for predicting AHI ≥5/h, ≥15/h, and ≥30/h, respectively (**Table 2**, **Figure 2**). The sensitivity of ODI ≥5/h, ≥15/h, and ≥25/h was 92%, 90%, and 93% for AHI ≥5/h, ≥15/h, and ≥30/h, respectively, while the specificity was 73%, 89%, and 85%, respectively (**Table 2**). Results of AUCs, sensitivity, specificity, negative and positive predictive values with the use of the full data set also showed good diagnostic performance (**Supplementary Figure S2**, and **Figure S3**).

**Table 2:** Diagnostic accuracy of oxygen desaturation index in classifying sleep-disordered breathing categories across three cutoffs.

	Derivation dataset, % (95% CI)	Validation dataset, % (95% CI)
**AHI ≥5/h**		
Sensitivity	98 (95–99)	92 (86–96)
Specificity	74 (53–88)	73 (45–91)
PPV	97 (94–99)	97 (92–99)
NPV	77 (56–90)	52 (30–74)
AUC	95.6 (91.7–99.4)	94.1 (90.4–97.8)
**AHI ≥15/h**		
Sensitivity	93 (88–96)	90 (82–95)
Specificity	84 (75–90)	89 (76–95)
PPV	90 (85–94)	93 (86–97)
NPV	88 (80–93)	84 (71–92)
AUC	96.2 (94.3–98.1)	96.9 (94.7–99.1)
**AHI ≥30/h**		
Sensitivity	97 (92–99)	93 (82–98)
Specificity	86 (80–90)	85 (75–91)
PPV	82 (75–88)	78 (66–87)
NPV	98 (94–99)	95 (87–98)
AUC	97.6 (95.6–99.6)	94.9 (91.5–98.3)

AHI, apnea-hypopnea index; CI, confidence interval; PPV, positive predictive value; NPV, negative predictive value; AUC, area under the receiver operating characteristic curve.

For each AHI cutoff, different ODIs were derived for prediction. 5/h, 15/h, and 25/h was derived to assess the diagnostic accuracy across three AHI cutoffs (5/h, 15/h, and 30/h).

## Discussion

This study showed that ODI had a strong correlation with AHI in patients with diabetes. ODI had a very high accuracy to predict SDB, with the AUC of 0.941, 0.969, and 0.949 for AHI ≥5/h, ≥15/h, and ≥30/h, respectively, in the validation group. The sensitivity of ODI ≥5/h, ≥15/h, and ≥25/h was 92%, 90%, and 93%, respectively for predicting AHI ≥5/h, ≥15/h, and ≥30/h, while the specificity was 73%, 89%, and 85%, respectively.

To our knowledge, this is the first study to assess the diagnostic accuracy of ODI for SDB in patients with diabetes. The high prevalence of SDB in patients with diabetes and the bidirectional association make it necessary to screen SDB in patients with diabetes (5, 17, 25). However, because of the lack of a convenient and inexpensive tool, it has been a challenge to precisely identify patients with SDB in the diabetes population in clinical practice. The results of this study suggested that ODI could be a useful tool to assist recognizing patients with SDB. The high sensitivity of 93% of ODI ≥5/h enables us to exclude the possibility of SDB if a patient with diabetes had an ODI ≤5/h. Thus, utility of ODI could reduce the number of PSG tests.

On the other hand, the specificity of ODI ≥15/h and ODI ≥25/h to predict moderate and severe SDB was as high as 86% and 94%, respectively. Therefore, we would be confident to consider a patient with an ODI ≥15/h as having moderate to severe SDB, and a patient with ODI ≥25/h as having severe SDB. Given the high prevalence of SDB in patients with diabetes (4), an ODI more than 25/h would have a high positive predictive value of 83% for severe SDB, as stated in this study. This would prompt clinicians to take measures and avoid delayed detection and therapy of severe SDB for those patients with high ODI, particularly in situations where waiting list for PSG is too long.

Using nocturnal oximetry to screen patients with SDB has been exploited in the general population in previous studies. However, significant heterogeneity exists with sensitivities ranged from 32% to 98.5%, whereas specificities ranged from 47.7% to 98% (18). Chiner et al., Lin et al., Hang et al. found that ODI was a good predictor for AHI, with sensitivities more than 84.0% and specificities more than 87.8% for predicting SDB (26–28). Chung et al. assessed the predictive value of ODI in patients who were scheduled for inpatient surgery for SDB. They found that ODI was a good predictor for AHI, with AUC for ODI to predict AHI ≥5/h, ≥15/h, and ≥30/h, was 0.908 (95%CI 0.880–0.936), 0.931 (95%CI 0.090–0.952), and 0.958 (95%CI 0.937–0.979), respectively (29). These results are consistent with the high diagnostic accuracy of our study.

However, other studies did not found that ODI provided a satisfactory diagnostic accuracy. Alvarez et al., Golpe et al., and Gyulay et al. found that ODI had a high specificity of 94.3%, 97%, and 98%, with a low sensitivity of 58.7%, 32%, and 40%, respectively (30–32). The high specificity suggested that ODI was a better rule in tool, rather than a rule out tool. Several possible reasons can be put forward to explain these discrepancies. First, in our study, the pre-test probability of SDB in patients with diabetes is higher than that in the general population. This might lead to the relatively high diagnostic accuracy. Second, the population heterogeneity might contribute the difference of the sensitivity and specificity of ODI, because the accuracy of ODI lies in the detection of apnea while hypopnea are underdiagnosed (78% of apneas and 54% hypopneas caused desaturation) (33). Third, different oximeters might have great difference in calculating the number of desaturations. Fourth, Different criteria for grading SDB severity and different definitions of hypopnea, oxygen desaturation, baseline airflow and baseline saturation might also account for the variations.

In our study, neither the inclusion of Epworth score nor excessive daytime sleepiness (Epworth score ≥10) had better predictive effect than ODI alone. Furthermore, the predictive value of Epworth score for SDB was very low with the AUC between 0.5 and 0.6 (**Supplementary Table S5**). This was consistent with previous studies (10, 12). It may be because that sleepiness is not necessarily concordant with SDB severity.

There are a few limitations in this study. First, due to the relatively high age (average age 58 ± 14 years) and high prevalence of commodities of the patients with diabetes in this study, further research should be done to verify whether the results could be generalized to the whole diabetes population. Second, the prevalence of SDB in this study was as high as 90.5%, this may affect the positive and negative predictive value of ODI. However, the sensitivities and specificities should not be affected. Third, the prediction of ODI was based on the AHI cutoffs. Although AHI was regarded as the diagnostic standard of SDB, AHI was not the best predictor of SDB-related morbidity (34). In the future, the morbidity and outcomes, such as cardiovascular mortality, should be considered as the targets of prediction. Fourth, Chronic obstructive pulmonary disease (COPD) or other pulmonary disorders which could influence the oxygen desaturation was not considered in this study. Fifth, although ODI is a good predictor of SDB, it only reflects the oxygen saturation change and does not monitor nasal flow and respiratory effort. Thus, it is not able to distinguish obstructive sleep apnea and central sleep apnea.

The results in our study have implications for clinical practice. The high diagnostic accuracy of ODI for SDB suggests that ODI could be used to reduce the number of PSG test for those with low ODI, and avoid delayed detection and therapy of severe SDB for those patients with high ODI. However, it is worth noting that for patients who are suffering from COPD or other pulmonary disorders, oximetry alone is not an appropriate method to predict SDB. What is more, because the oxygen signal is derived from the PSG system in this study, the predictive activity of ODI has to be validated in pulse oximetry devices. The loss of signal during test of oxygen signal should also be paid attention during clinical practice. Furthermore, in addition to pulse oximetry, oxygen saturation metrics could be derived from other accessible devices, such as smartphones and wearable devices (35). Thus, a simple overnight screening and everyday monitoring could become possible. However, these devices are often not certified to give medical accurate information on oxygen saturation (36). Signal quality, signal treatment, and averaging over several pulses may vary and differ largely compared to oximetry integrated in a polysomnography as used here. Thus, the accuracy of these devices should be evaluated carefully before they can be used to make medical predictions.

## Conclusion

In conclusion, ODI was strongly correlated with AHI in patients with diabetes. Based on maximal accuracy and simplicity of clinical use, ODI ≥5/h, ODI ≥15/h, ODI ≥25/h were good predictors for mild, moderate, and severe SDB for patients with diabetes, respectively. Further studies should be carried out to validate the diagnostic accuracy of ODI in the community-based diabetes population for greater clinical application.

## Data Availability Statement

The original contributions presented in the study are included in the article/**Supplementary Material**. Further inquiries can be directed to the corresponding author.

## Ethics Statement

The studies involving human participants were reviewed and approved by Biomedical Research Ethics Committee of West China Hospital of Sichuan University. The patients/participants provided their written informed consent to participate in this study.

## Author Contributions

LC and WT designed the study, collected data, did statistical analysis, and drafted the manuscript. CW, DC, YG, WM, and PZ designed the study and collected data and contributed to the statistical analysis. FL and XT participated in the study design, data collection and data analysis. XR designed and coordinated the study, acquired funding, and participated in writing and editing the final manuscript. All authors contributed to the article and approved the submitted version.

## Funding

This study was supported by National Science and Technology Major Project (grant no. 2017ZX09304023), Science and Technology Bureau of Chengdu city (grant no 2017-CY02-00028-GX), Health Medical Big Data Application and Innovation Project in Sichuan (grant no. 2018gfgw001), 1.3.5 Project for disciplines of excellence, West China Hospital, Sichuan University (grant no. ZYGD18025), and National Key R&D Program of China (grant no. 2017YFC1309605).

## Conflict of Interest

The authors declare that the research was conducted in the absence of any commercial or financial relationships that could be construed as a potential conflict of interest.

## References

[1] Dempsey JA Veasey SC Morgan BJ O’Donnell CP. Pathophysiology of sleep apnea. Physiol Rev (2010) 90(1):47–112. 10.1152/physrev.00043.2008 20086074 PMC3970937

[2] Peppard PE Young T Barnet JH Palta M Hagen EW Hla KM. Increased prevalence of sleep-disordered breathing in adults. Am J Epidemiol (2013) 177(9):1006–14. 10.1093/aje/kws342 PMC3639722 23589584

[3] Tahrani AA. Obstructive Sleep Apnoea and Vascular Disease in Patients with Type 2 Diabetes. Eur Endocrinol (2015) 11(2):81–9. 10.17925/EE.2015.11.02.81 PMC5819072 29632575

[4] Zhang P Zhang R Zhao F Heeley E Chai-Coetzer CL Liu J. The prevalence and characteristics of obstructive sleep apnea in hospitalized patients with type 2 diabetes in China. J Sleep Res (2016) 25(1):39–46. 10.1111/jsr.12334 26268508

[5] Chen L Ma W Tang W Zha P Wang C Chen D. Prevalence of obstructive sleep apnea in patients with diabetic foot ulcers. Front Endocrinol (2020) 11:416. 10.3389/fendo.2020.00416 PMC7371781 32760345

[6] Vianello A Bisogni V Rinaldo C Gallan F Maiolino G Braccioni F. Recent advances in the diagnosis and management of obstructive sleep apnea. Minerva Med (2016) 107(6):437–51. 27625198

[7] Tahrani AA Ali A Raymond NT Begum S Dubb K Mughal S. Obstructive sleep apnea and diabetic neuropathy: a novel association in patients with type 2 diabetes. Am J Respir Crit Care Med (2012) 186(5):434–41. 10.1164/rccm.201112-2135OC PMC3443800 22723291

[8] Schaefer CA Adam L Weisser-Thomas J Pingel S Vogel G Klarmann-Schulz U. High prevalence of peripheral arterial disease in patients with obstructive sleep apnoea. Clin Res Cardiol (2015) 104(9):719–26. 10.1007/s00392-015-0834-3 25725776

[9] Kapur VK Auckley DH Chowdhuri S Kuhlmann DC Mehra R Ramar K. Clinical Practice Guideline for Diagnostic Testing for Adult Obstructive Sleep Apnea: An American Academy of Sleep Medicine Clinical Practice Guideline. J Clin Sleep Med (2017) 13(3):479–504. 10.5664/jcsm.6506 28162150 PMC5337595

[10] Singla V Gattu T Aggarwal S Bhambri A Agarwal S. Evaluation of Epworth Sleepiness Scale to Predict Obstructive Sleep Apnea in Morbidly Obese Patients and Increasing Its Utility. J Laparoendosc Adv Surg Tech A (2019) 29(3):298–302. 10.1089/lap.2018.0329 30109974

[11] Martins EF Martinez D Cortes AL Nascimento N Brendler J. Exploring the STOP-BANG questionnaire for obstructive sleep apnea screening in seniors. J Clin Sleep Med (2020) 16(2):199–206. 10.5664/jcsm.8166 31992408 PMC7053028

[12] Chiu HY Chen PY Chuang LP Chen NH Tu YK Hsieh YJ. Diagnostic accuracy of the Berlin questionnaire, STOP-BANG, STOP, and Epworth sleepiness scale in detecting obstructive sleep apnea: A bivariate meta-analysis. Sleep Med Rev (2017) 36:57–70. 10.1016/j.smrv.2016.10.004 27919588

[13] Jonas DE Amick HR Feltner C Weber RP Arvanitis M Stine A. Screening for Obstructive Sleep Apnea in Adults: Evidence Report and Systematic Review for the US Preventive Services Task Force. JAMA (2017) 317(4):415–33. 10.1001/jama.2016.19635 28118460

[14] Kirby SD Eng P Danter W George CF Francovic T Ruby RR. Neural network prediction of obstructive sleep apnea from clinical criteria. Chest (1999) 116(2):409–15. 10.1378/chest.116.2.409 10453870

[15] Huang WC Lee PL Liu YT Chiang AA Lai FP. Support Vector Machine Prediction of Obstructive Sleep Apnea in a Large-Scale Chinese Clinical Sample. Sleep (2020) 43(7):zsz295. 10.1093/sleep/zsz295 31917446 10.1093/sleep/zsz295 PMC7355399

[16] Zhang Z Beck MW Winkler DA Huang B Sibanda W Goyal H. Opening the black box of neural networks: methods for interpreting neural network models in clinical applications. Ann Transl Med (2018) 6(11):216. 10.21037/atm.2018.05.32 30023379 PMC6035992

[17] Subramanian A Adderley NJ Tracy A Taverner T Hanif W Toulis KA. Risk of Incident Obstructive Sleep Apnea Among Patients With Type 2 Diabetes. Diabetes Care (2019) 42(5):954–63. 10.2337/dc18-2004 30862657

[18] Rashid NH Zaghi S Scapuccin M Camacho M Certal V Capasso R. The Value of Oxygen Desaturation Index for Diagnosing Obstructive Sleep Apnea: A Systematic Review. Laryngoscope (2021) 131(2):440–7. 10.1002/lary.28663 32333683

[19] Mazzotti DR Keenan BT Lim DC Gottlieb DJ Kim J Pack AI. Symptom Subtypes of Obstructive Sleep Apnea Predict Incidence of Cardiovascular Outcomes. Am J Respir Crit Care Med (2019) 200(4):493–506. 10.1164/rccm.201808-1509OC 30764637 PMC6701040

[20] Malhotra A Orr JE Owens RL. On the cutting edge of obstructive sleep apnoea: where next? Lancet Respir Med (2015) 3(5):397–403. 10.1016/S2213-2600(15)00051-X 25887980 PMC4431916

[21] Neumann C Martinez D Schmid H. Nocturnal oxygen desaturation in diabetic patients with severe autonomic neuropathy. Diabetes Res Clin Pract (1995) 28(2):97–102. 10.1016/0168-8227(95)01053-G 7587925

[22] Johns M Hocking B. Daytime sleepiness and sleep habits of Australian workers. Sleep (1997) 20(10):844–9. 10.1093/sleep/20.10.844 9415943

[23] Berry RB Budhiraja R Gottlieb DJ Gozal D Iber C Kapur VK. Rules for scoring respiratory events in sleep: update of the 2007 AASM Manual for the Scoring of Sleep and Associated Events. Deliberations of the Sleep Apnea Definitions Task Force of the American Academy of Sleep Medicine. J Clin Sleep Med JCSM (2012) 8(5):597–619. 10.5664/jcsm.2172 23066376 PMC3459210

[24] Hawass NE. Comparing the sensitivities and specificities of two diagnostic procedures performed on the same group of patients. Br J Radiol (1997) 70(832):360–6. 10.1259/bjr.70.832.9166071 9166071

[25] Ma W Chen L Tang X Ran X. Obstructive sleep apnea and diabetic foot: a novel research target? Clin Focus (2019) 34(8):4. 10.3969/j.issn.1004-583X.2019.08.001

[26] Chiner E. Nocturnal oximetry for the diagnosis of the sleep apnoea hypopnoea syndrome: a method to reduce the number of polysomnographies? Thorax (1999) 54:968–71. 10.1136/thx.54.11.968 PMC1745404 10525553

[27] Lin CL Yeh C Yen CW Hsu WH Hang LW. Comparison of the indices of oxyhemoglobin saturation by pulse oximetry in obstructive sleep apnea hypopnea syndrome. Chest (2009) 135(1):86–93. 10.1378/chest.08-0057 18689584

[28] Hang L-W Wang H-L Chen J-H Hsu J-C Lin H-H Chung W-S. Validation of overnight oximetry to diagnose patients with moderate to severe obstructive sleep apnea. BMC Pulmonary Med (2015) 15(1). 10.1186/s12890-015-0017-z PMC4407425 25880649

[29] Chung F Liao P Elsaid H Islam S Shapiro CM Sun Y. Oxygen desaturation index from nocturnal oximetry: a sensitive and specific tool to detect sleep-disordered breathing in surgical patients. Anesth Analg (2012) 114(5):993–1000. 10.1213/ANE.0b013e318248f4f5 22366847

[30] Alvarez D Hornero R Garcia M del Campo F Zamarron C. Improving diagnostic ability of blood oxygen saturation from overnight pulse oximetry in obstructive sleep apnea detection by means of central tendency measure. Artif Intell Med (2007) 41(1):13–24. 10.1016/j.artmed.2007.06.002 17643971

[31] Golpe R. Utility of Home Oximetry as a Screening Test for Patients with Moderate to Severe Symptoms of Obstructive Sleep Apnea. Sleep (1999). 22(7):932–7 10566911

[32] Gyulay S Olson LG Hensley MJ King MT Allen KM Saunders NA. A comparison of clinical assessment and home oximetry in the diagnosis of obstructive sleep apnea. Am Rev Respir Dis (1993) 147(1):50–3. 10.1164/ajrccm/147.1.50 8420431

[33] Ayappa I Rapaport BS Norman RG Rapoport DM. Immediate consequences of respiratory events in sleep disordered breathing. Sleep Med (2005) 6(2):123–30. 10.1016/j.sleep.2004.08.005 15716216

[34] Zinchuk A Yaggi HK. Sleep Apnea Heterogeneity, Phenotypes, and Cardiovascular Risk. Implications for Trial Design and Precision Sleep Medicine. Am J Respir Crit Care Med (2019) 200(4):412–3. 10.1164/rccm.201903-0545ED PMC6701039 30916985

[35] Garde A Dekhordi P Ansermino JM Dumont GA. Identifying individual sleep apnea/hypoapnea epochs using smartphone-based pulse oximetry. Annu Int Conf IEEE Eng Med Biol Soc (2016) 2016:3195–8. 10.1109/EMBC.2016.7591408 28268987

[36] MM A DK R Li J Scott JB. Reliability of Smartphone Pulse Oximetry in Subjects at Risk for Hypoxemia. Respir Care (2020) 66(3):384–90. 10.4187/respcare.07670 33023999

