# Supplementary material for: Diagnostic Accuracy of Oxygen Desaturation Index for Sleep-Disordered Breathing in Patients With Diabetes
Source: Front Endocrinol (Lausanne). 2021 Mar 9;12:598470. doi: 10.3389/fendo.2021.598470 (PMC7985532; doi:10.3389/fendo.2021.598470)
Supplement: Supplementary file 1 [file DataSheet_1.docx]

**Supplementary Appendix**

**Diagnostic accuracy of oxygen desaturation index for sleep-disordered breathing**

**in patients with diabetes**

Lihong Chen, Weiwei Tang, Chun Wang, Dawei Chen, Yun Gao, Wanxia Ma, Panpan Zha, Fei Lei, Xiangdong Tang, Xingwu Ran

| **Table of contents** | **Page** |
| --- | --- |
| **Table S1. Baseline characteristics of patients in the derivation dataset across three AHI cutoffs** | **2** |
| **Table S2. Baseline characteristics of patients in the validation dataset across three AHI cutoffs** | **3** |
| **Table S3. Comparison of AUCs among three models in the derivation dataset** | **4** |
| **Table S4. Comparison of sensitivity and specificity between derived cutoffs and simplified cutoffs for classifying sleep-disordered breathing across three categories** | **5** |
| **Table S5.** **Predictive performance of Epworth score for sleep-disordered breathing** | **6** |
| **Figure S1. Apnea hypopnea index (AHI) versus oxygen desaturation index (ODI) with linear regression and 95% confidence interval** | **7** |
| **Figure S2. Oxygen desaturation index in the full dataset at three AHI cutoffs (Panel A: AHI ≥5/h, Panel B: AHI ≥15/h, and Panel C: AHI ≥30/h)** | **8** |
| **Figure S3. Predictive performance of oxygen desaturation index for sleep-disordered breathing in the full dataset at three cutoffs (Panel A: 5/h, Panel B: 15/h, and Panel C: 30/h)** | **9** |

**Table S1. Baseline characteristics of patients in the derivation dataset across three AHI cutoffs**

|  | Total | AHI <5 | AHI >=5 | AHI <15 | AHI >=15 | AHI <30 | AHI >=30 |
| --- | --- | --- | --- | --- | --- | --- | --- |
|  | N =293 | N =27 | N =266 | N =111 | N =182 | N =175 | N =118 |
| Male sex | 191(65%) | 13(48%) | 178(67%) | 72(65%) | 119(65%) | 120(69%) | 71(60%) |
| Age, y/o | 57±14 | 51±16 | 58±14* | 55±15 | 58±14 | 56±14 | 58±15 |
| BMI, kg/m^2^ | 26.0±6.1 | 24.7±5.7 | 26.1±6.1 | 24.4±6.6 | 27.0±5.5^&^ | 25.0±6.0 | 27.5±5.9^#^ |
| Smoking | 137(47%) | 7(26%) | 130(49%)* | 54(49%) | 83(46%) | 87(50%) | 50(42%) |
| Duration of diabetes, y | 9(3-16) | 4(1-10) | 10(3-17)^&^ | 7(2-12) | 10(3-18)* | 8(3-13) | 10(3-18) |
| Hypertension | 190(65%) | 8(30%) | 182(68%)^#^ | 53(48%) | 137(75%)^#^ | 100(57%) | 90(76%)^&^ |
| Ischemic heart disease | 46(16%) | 3(11%) | 43(16%) | 18(16%) | 28(15%) | 26(15%) | 20(17%) |
| Peripheral artery disease | 49(17%) | 2(7%) | 47(18%) | 19(17%) | 30(16%) | 31(18%) | 18(15%) |
| Diabetic Retinopathy | 78(27%) | 6(22%) | 72(27%) | 25(23%) | 53(29%) | 42(24%) | 36(31%) |
| Peripheral nervous disease | 188(64%) | 17(63%) | 171(64%) | 81(73%) | 107(59%)* | 122(70%) | 66(56%)* |
| Diabetic kidney disease | 92(31%) | 7(26%) | 85(32%) | 35(32%) | 57(31%) | 52(30%) | 40(34%) |
| Diabetic foot ulcer | 79(27%) | 5(19%) | 74(28%) | 36(32%) | 43(24%) | 54(31%) | 25(21%) |
| HbA1c, % | 8.7±2.2 | 8.5±2.1 | 8.7±2.2 | 8.9±2.2 | 8.5±2.2 | 8.9±2.2 | 8.2±2.1 |
| Epworth score | 6(2-10) | 4.5(2-10) | 6(2-10) | 5(2-9) | 6(2-11) | 5(2-9) | 7(3-13) * |
| >10 | 89(30%) | 9(33%) | 80(30%) | 32(29%) | 57(31%) | 44(25%) | 45(38%)* |
| AHI,/h | 24.0(9.7-43.6) | 2.6(1.1-3.5) | 26.3(12.1-46.8) ^#^ | 8.0(5.1-10.6) | 36.8(25.6-53.1) ^#^ | 11.1(6.6-18.5) | 48.9(37.2-62.7) ^#^ |
| Total sleep time, h | 6.5±1.6 | 6.5±1.3 | 6.5±1.7 | 6.4±1.5 | 6.6±1.7 | 6.5±1.6 | 6.7±1.7 |
| Sleep efficiency, % | 74±15 | 74±14 | 74±14 | 73±15 | 75±16 | 74±15 | 75±16 |
| T90%, % | 4(1-16) | 0(0-8) | 5(1-17) ^#^ | 0(0-4) | 8(3-23) ^#^ | 1(0-7) | 14(5-31) ^#^ |
| Mean oxygen saturation, % | 93±4 | 94±3 | 93±4 | 94±2 | 92±5^#^ | 94±2 | 92±6^#^ |
| Oxygen desaturation index, /h | 22.5(10.9-42.4) | 2.8(1.4-5.4) | 26.2(12.8-45.5) ^#^ | 9.1(5.7-12.7) | 36.1(25.2-54.1) ^#^ | 12.7(7.4-20) | 49.3(36.8-64.7) ^#^ |

Data are shown as mean (SD), median (quartile), or count (%). BMI, body mass index; AHI, apnea-hypopnea index; HbA1c, hemoglobin A1c; T90%, percentage of time with oxygen saturation < 90%. *, <0.05; &, <0.01; #, <0.001.

**Table S2. Baseline characteristics of patients in the validation dataset across three AHI cutoffs**

|  | Total | AHI <5 | AHI >=5 | AHI <15 | AHI >=15 | AHI <30 | AHI >=30 |
| --- | --- | --- | --- | --- | --- | --- | --- |
|  | N =147 | N =15 | N =132 | N =53 | N =94 | N =92 | N =55 |
| Male sex | 99(67%) | 10(67%) | 89(67%) | 37(70%) | 62(66%) | 60(65%) | 39(71%) |
| Age, y/o | 59±14 | 54±17 | 59±14 | 58±13 | 60±15 | 59±13 | 59±15 |
| BMI, kg/m^2^ | 25.8±5.9 | 25.2±3.7 | 25.8±5.9 | 24.5±5.4 | 26.5±5.7 | 25.4±5.2 | 26.3±6.5 |
| Smoking | 73(50%) | 9(60%) | 64(48%) | 33(62%) | 40(43%)* | 45(49%) | 28(51%) |
| Duration of diabetes, y | 10(3-15) | 7(1-10) | 10(4-15) | 8(3-13) | 10(4-17) | 9.5(3.5-15) | 10(3-17) |
| Hypertension | 102(69%) | 9(60%) | 93(70%) | 33(62%) | 69(73%) | 61(66%) | 41(75%) |
| Ischemic heart disease | 27(18%) | 0(0%) | 27(20%) | 5(9%) | 22(23%)* | 12(13%) | 15(27%)* |
| Peripheral artery disease | 28(19%) | 1(7%) | 27(20%) | 10(19%) | 18(19%) | 21(23%) | 7(13%) |
| Retinopathy | 39(27%) | 2(13%) | 37(28%) | 13(25%) | 26(28%) | 24(26%) | 15(27%) |
| Peripheral nervous disease | 94(64%) | 10(67%) | 84(62%) | 34(64%) | 60(64%) | 60(65%) | 34(62%) |
| Nephropathy | 51(35%) | 9(60%) | 42(32%)* | 22(42%) | 29(31%) | 32(35%) | 19(35%) |
| Diabetic foot ulcer | 31(21%) | 5(30%) | 26(20%) | 13(25%) | 18(19%) | 21(23%) | 10(18%) |
| HbA1c, % | 8.9±2.2 | 9.2±2.3 | 8.8±2.2 | 9.0±2.4 | 8.8±2.2 | 9.0±2.5 | 8.8±1.9 |
| Epworth score | 6(3-11) | 6.5(3-8) | 6(2.5-11) | 6(3-8) | 7(2-12) | 6(2-10) | 7.5(3-12.5) |
| >10 | 51(35%) | 4(27%) | 47(36%) | 13(43%) | 38(40%) | 28(30%) | 23(42%) |
| AHI, /h | 21.0(9.8-38) | 3.7(1.3-4.4) | 25.6(12.9-41.7) ^#^ | 8.5(4.5-10.9) | 32.1(24.1-46.8) ^#^ | 12.2(6.9-19.5) | 44.2(35.0-62.2) ^#^ |
| Total sleep time, h | 6.4±1.6 | 6.1±1.5 | 6.5±1.6 | 6.5±1.6 | 6.4±1.7 | 6.3±1.7 | 6.7±1.5 |
| Sleep efficiency, % | 69±15 | 75±15 | 76±15 | 76±15 | 75±16 | 74±16 | 77±15 |
| T90%, % | 3(1-15) | 0(0-1) | 4(1-16) ^&^ | 1(0-3) | 6(0-17) ^#^ | 1(0-6) | 12(4-19) ^#^ |
| Mean oxygen saturation, % | 94±3 | 95±2 | 94±3* | 94±3 | 93±3* | 94±3 | 93±3^&^ |
| Oxygen desaturation index, /h | 21.3(10.9-35.2) | 4(1.7-5.7) | 24.6(13-36.4) ^#^ | 7(3.9-12.5) | 31.8(22.7-48) ^#^ | 12.6(5.9-20.4) | 41.1(31.5-58.1) ^#^ |

Data are shown as mean (SD), median (quartile), or count (%). BMI, body mass index; AHI, apnea-hypopnea index; HbA1c, hemoglobin A1c; T90%, percentage of time with oxygen saturation < 90%. *, <0.05; &, <0.01; #, <0.001.

**Table S3. Comparison of AUCs among three models in the derivation dataset**

| AHI | Model 1 | Model 2 | Model 3 | P value |
| --- | --- | --- | --- | --- |
| ≥ 5/h | 0.946 (0.897-0.992) | 0.947 (0.901-0.993) | 0.969 (0.947-0.991) | 0.394 |
| ≥ 15/h | 0.963 (0.944-0.983) | 0.965 (0.946-0.984) | 0.969 (0.951-0.987) | 0.226 |
| ≥ 30/h | 0.980 (0.962-0.998) | 0.981 (0.962-0.999) | 0.984 (0.965-1.000) | 0.441 |

Model 1, only oxygen desaturation index; Model 2, Epworth score, and oxygen desaturation index; Model 3, sex, age, BMI, Epworth score, mean oxygen saturation, and oxygen desaturation index. AHI, apnea-hypopnea index.

Comparison of AUCs among models were after logistic linear predictors.

**Table S4. Comparison of sensitivity and specificity between derived cutoffs and simplified cutoffs for classifying sleep-disordered breathing across three categories**

|  | Derived cutoffs, % (95%CI) | Simplified cutoffs, % (95%CI) | P value |
| --- | --- | --- | --- |
| **AHI ≥5/h (7.4 vs. 5)** |  |  |  |
| Sensitivity | 92 (88-95) | 98 (95-99) | 0.001 |
| Specificity | 89 (70-97) | 74 (53-88) | 0.134 |
| **AHI ≥15/h (16.8 vs. 15)** |  |  |  |
| Sensitivity | 90 (85-94) | 93 (88-96) | 0.074 |
| Specificity | 90 (83-95) | 84 (75-90) | 0.023 |
| **AHI ≥30/h (26.8 vs. 25)** |  |  |  |
| Sensitivity | 96 (90-98) | 97 (92-99) | 0.480 |
| Specificity | 91 (86-95) | 86 (80-90) | 0.008 |

Derived cutoff across SDB categories were 7.4/h, 16.8h, and 26.8/h; simplified cutoff were 5/h, 15/h, 25/h. AHI, apnea-hypopnea index.

**Table S5. Predictive performance of Epworth score for sleep-disordered breathing**

|  | Full dataset, %(95%CI) |
| --- | --- |
| **AHI ≥5/h** |  |
| Sensitivity | 32 (27-37) |
| Specificity | 69 (53-82) |
| PPV | 91 (84-95) |
| NPV | 10 (7-14) |
| AUC | 52.0 (42.6-61.5) |
| **AHI ≥15/h** |  |
| Sensitivity | 34 (29-40) |
| Specificity | 73 (65-79) |
| PPV | 68 (59-75) |
| NPV | 40 (34-45) |
| AUC | 52.6 (47.0-58.3) |
| **AHI ≥30/h** |  |
| Sensitivity | 39 (32-47) |
| Specificity | 73 (67-78) |
| PPV | 49 (40-57) |
| NPV | 65 (59-70) |
| AUC | 57.7 (52.1-63.4) |

AHI, apnea-hypopnea index; CI, confidence interval; PPV, positive predictive value; NPV, negative predictive value; AUC, area under the receiver operating characteristic curve.

Epworth Score ≥10 was used to assess the diagnostic accuracy (sensitivity, specificity, PPV, and NPV) across three AHI cutoffs (5/h, 15/h, and 30/h).


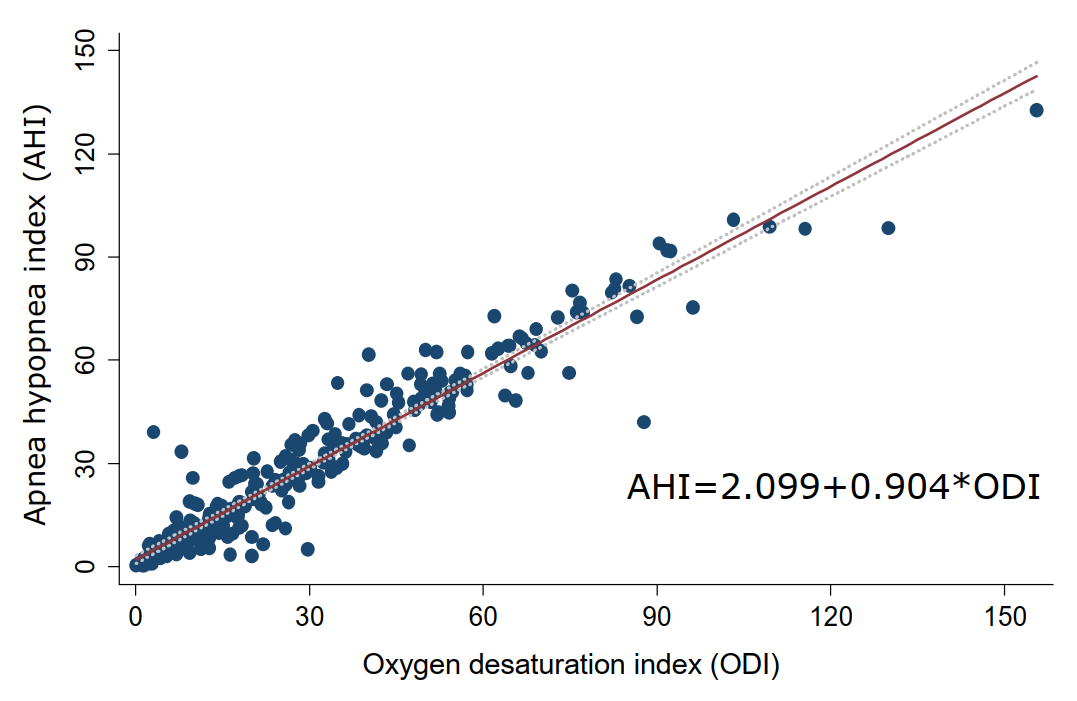


**Figure S1. Apnea hypopnea index (AHI) versus oxygen desaturation index (ODI) with linear regression and 95% confidence interval.** R^2^ = 0.920, AHI =2.099+0.904*ODI.


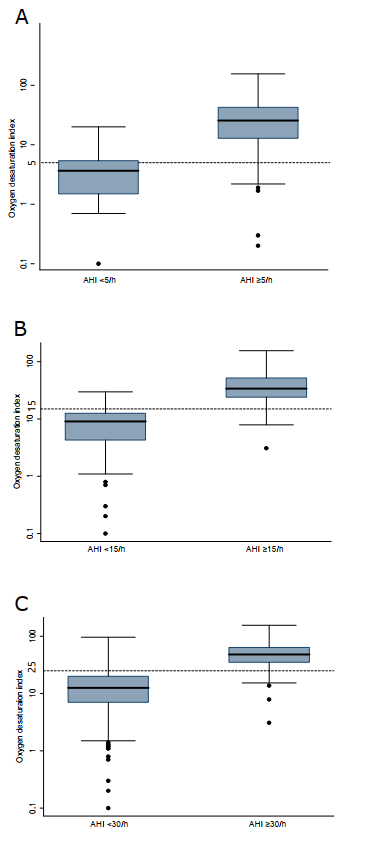


**Figure S2. Oxygen desaturation index in the full dataset at three AHI cutoffs (Panel A: AHI ≥5/h, Panel B: AHI ≥15/h, and Panel C: AHI ≥30/h)**. The bottom and top boundaries of each box represent the upper and lower quartiles, the lines within the box represent the median, the whiskers represent values that are 1.5 times the interquartile range, the dots represent values outside the range, and the horizontal lines represents the cutoff point of Oxygen desaturation index.


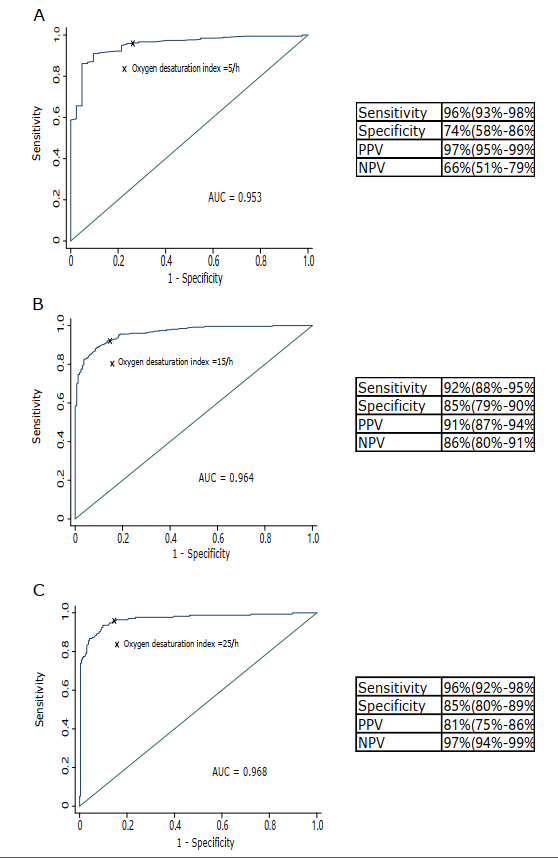


**Figure S3. Predictive performance of oxygen desaturation index for sleep-disordered breathing in the full dataset at three cutoffs (Panel A: 5/h, Panel B: 15/h, and Panel C: 30/h)**.
